# Sun exposure to the eyes: predicted UV protection effectiveness of various sunglasses

**DOI:** 10.1038/s41370-018-0087-0

**Published:** 2018-10-31

**Authors:** C. Backes, A. Religi, L. Moccozet, F. Behar-Cohen, L. Vuilleumier, J. L. Bulliard, D. Vernez

**Affiliations:** 1 Institute for Work and Health, University of Lausanne and Geneva, 1010 Lausanne, Switzerland; 2 Division of Chronic Diseases, University Institute of Social and Preventive Medicine, Lausanne University Hospital (CHUV), Lausanne, Switzerland; 3 Centre Universitaire d’Informatique, University of Geneva, Geneva, Switzerland; 4 INSERM U1138, Centre de Recherche des Cordeliers, Université Paris Descartes, Université Pierre et Marie Curie, Paris, France; 5 Federal Office of Meteorology and Climatology (MeteoSwiss), Payerne, Switzerland

## Abstract

The aim of this study was to assess solar ultraviolet radiation (UVR) doses received by the eyes in different exposure situations, and to predict the sun protection effectiveness provided by various styles of sunglasses at facial, periorbital, and ocular skin zones including the cornea and accounting for different head positions. A 3D numeric model was optimized to predict direct, diffuse and reflected erythemally weighted UVR doses received at various skin zones. Precisely defined facial, periorbital, and ocular skin zones, sunglasses (goggles, medium-, and large-sized sunglasses) and three head positions were modeled to simulate daily (08:00–17:00) and midday (12:00–14:00) UVR doses. The shading from sunglasses’ frame and lenses’ UVR transmission were used to calculate a predictive protection factor (PPF [%]). Highest ocular daily UVR doses were estimated at the uncovered cornea (1718.4 J/m^2^). Least sun protection was provided by middle-sized sunglasses with highest midday dose at the white lateral (290.8 J/m^2^) and lateral periorbital zones (390.9 J/m^2^). Goggles reached almost 100% protection at all skin zones. Large-sized sunglasses were highly effective in winter; however, their effectiveness depended on diffuse UVR doses received. In “looking-up” head positions highest midday UVR doses were received at the unprotected cornea (908.1 J/m^2^), totally protected when large-sized sunglasses are used. All tested sunglass lenses fully blocked UVR. Sunglasses’ protection effectiveness is strongly influenced by geometry, wearing position, head positions, and exposure conditions. Sunglasses do not totally block UVR and should be combined with additional protection means. 3D modeling allows estimating UVR exposure of highly sensitive small skin zones, chronically exposed and rarely assessed.

## Introduction

Ocular diseases, including cataract, eyelid malignancies, uveal melanoma, photokeratitis, droplet keratopathy, and macular degeneration are triggered by exposure to solar ultraviolet radiation (UVR) and visible blue light [1–5]. Evidence shows strong correlation between UVR exposure and skin and eye diseases [3, 6, 7]. However, their dose–response relationships remain poorly elucidated. Chronically exposed to sunlight, the quantification of the eye’s cumulative UVR doses received is crucial to understand the dose–response dependency and provide effective sun protection messages.

Since decades prevention campaigns have raised awareness about sun exposure hazards, aiming at reducing the UVR dose received by behavioral changes such as seeking shade, wearing a hat, sunglasses or long sleeves [8]. Sunglasses provide a vertical protection barrier to the eyes, whose effectiveness depends highly on the sunglasses lenses’ radiation transmittance, sunglasses geometry and the exposure conditions. Sunglasses’ protection effectiveness is generally communicated by non-harmonized sunglasses categories based on the lenses only, classifying the entire sun spectrum (UVR, infrared (IR) transmission and visible (Vis)) provided, without accounting for periorbital skin zones or environmental factors [9, 10].

High UVR doses can reach the eyes even when sunglasses are worn. Sun radiation reaches the eyes from above due to direct radiation circumventing the sunglasses, from below by reflected radiation from ground surfaces and from all other directions due to diffuse radiation from scattering by clouds and particles [11, 12]. An ineffective use of sunglasses might even increase UVR doses received due to pupils dilatation or prolonged outdoor exposure presuming a total ocular protection while wearing sunglasses [13].

The eye’s UVR absorption depends on the tissues considered and the person’s age and the wavelength received (UVB 280–315 nm or UVA 315–400 nm) [14, 15]. Before the age of 8–10 years, 2–5% of UVR received by the eyes can reach the retina, while over the age of 25 years, this will only be 1–2%. As most UVR doses received below 300 nm wavelengths (almost all UVB) are blocked by the cornea and the periorbital skin zones, the assessment of solar UVR doses received by these skin zones is crucial. Dose assessment by dosimeters is costly, time-consuming, context-specific, prone to behavioral bias, and their output is unable to distinguish between direct, diffuse, and reflected UVR; however, identified important influencing factors as effects of orientation toward the sun and geometry [16, 17]. Ultraviolet radiation reaching the surface of the eye has been measured by contact lens dosimetry and highlighted artefacts due to rotation and scratches [18]. Recently a substantial contribution of solar diffuse UVR to the total solar UVR received has been reported, a fact probably underestimated in public health messages [19]. Thus, the UVR dose estimation received by the periorbital skin region and the cornea, following the same reasoning as for other skin zones, is very complex to assess and often disregarded in prevention messages.

To address these issues, we used an improved version of a validated three-dimensional (3D) numeric model (SimUVEx) taking into account each solar UVR component (direct, diffuse, and reflected UVR) [20–22]. This study aims (1) at measuring sunglass lenses UVR transmission by dosimetry, (2) at estimating daily (08:00–17:00) solar erythemally weighted UVR doses received at different facial, periorbital and ocular skin zones including the cornea of a 3D head-form protected with or without various styles of sunglasses, (3) at predicting sunglasses sun protection effectiveness and (4) at analyzing head positions influence toward UVR doses received.

## Material and Methods

### Erythemally weighted UVR transmittance of sunglass lenses

In an experimental setup, the erythemally weighted UVR transmittance of three tinted (blue, brown, and green), commercially available, non-polarized plastic, category 3 (EN 1836: 2005) sunglass lenses was measured using as solar UVR source a 1000 W Xenon lamp (Solar LIGHT® Model LS-1000-4S-009 Solar Simulator, Glenside, Pennsylvania, USA) and a CIE erythemally weighted UVR dosimeter (Model X-2000 portable numeric dosimeter, Gigahertz-Optik GmbH, Puchheim, Germany) for the UVR transmittance [23]. The used Xenon lamp emits, besides infrared (IR) and visible (Vis) spectrum, a UVR spectrum comprised of 9.5% UVB and 90.5% UVA, mimicking the natural sunlight spectrum [24]. All three sunglass lenses used were new demonstration samples in excellent condition without any visible scratches. The experimental measurement setup ensured that no other UVR reached the dosimeter.

### Modeling tool

The predicted UVR doses potentially received by different skin zones were estimated by SimUVEx v.2 (Simulating UV Exposure version 2.0). This model uses ambient irradiance data, 3D human body modeling and computer graphics techniques, to estimate erythemally weighted solar UVR doses received at the skin within minutes. Its principles and validation with on-field dosimetry measurements have been detailed and published previously [20–22]. The model used for this study in not available as open source. In case of interest in collaborating, please contact the research group.

**Fig. 1:**
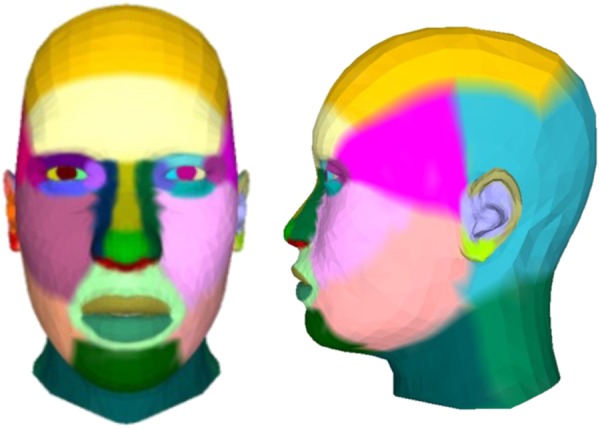
Front and side view of the head morphology with all delineated skin zones (total skin area: 3864 cm^2^) highlighted by specific colors. This study uses the following defined skin zones: the lower, upper and lateral periorbital skin zones and the tear ducts; the white lateral and medial ocular skin zones and the cornea, and the facial skin zones of the nose and the cheeks

For this study, the model was further developed. Well-defined facial, periorbital, and ocular skin zones were added to a detailed adult head morphology (Fig. 1). The following nine skin zones were considered: the facial skin zones of the nose and the cheeks, the periorbital skin zones divided by the lower, the upper and the lateral periorbital skin zones and the tear duct, and the ocular skin zones divided by the white lateral and white-medial ocular skin zones and the cornea.

The 3D head position was inclined by + 30°, 0°, and −30° angles from the horizontal to assess the influence of “looking up”, “looking straight forward”, and “looking down”, respectively. The tool allows static and dynamic functionalities with the latter obtained in mimicking the body movement through an azimuthal rotation between the simulation steps. A dynamic orientation with a 24° step rotation per minute was selected to simulate the head movements during a daily exposure.

### Input data

#### Ambient irradiance data

The model uses ambient irradiance as input data. Direct, diffuse and reflected UV erythemally weighted irradiance are measured at the MeteoSwiss Payerne Station (46.815°N, 6.944°E, altitude 491 m) using calibrated SolarLight SL 501A broadband radiometers with filters mimicking the erythema response. Radiometers measuring the direct and diffuse UVR components are mounted on sun following trackers. The radiometer measuring the direct component is enclosed in a collimating device excluding the diffuse irradiance outside a cone with a opening angle of about 5°. The radiometer measuring diffuse irradiance is shaded by a disk on a sun tracker arm, which ensures that irradiance from the sun direct beam is blocked. The radiometer measuring the reflected radiation is turned upside down. Irradiance data quality control procedures are conducted daily by using plausibility criteria on individual data components and by comparing the sum of direct and diffuse components to global UVR irradiance. UV radiometers are replaced every year by radiometers which went through a calibration check. This calibration check includes a 4-months comparison (between March and June, where ozone column is most variable) at Payerne with same type (SL501A) reference instruments, which are calibrated yearly at the World Radiation Center at Davos (PMOD/WRC). During the calibration check, the calibration constants and dependency matrices on ozone column and solar zenith angle are updated for each tested radiometer. Then the UV irradiances measured by each tested radiometer with the update calibration values are compared to those measured by the reference instruments, and radiometers are rejected and not used for the network if more than 5% irradiance data dissents by more than 5% from the mean of the corresponding irradiance of the reference radiometers. The Payerne station is part of the Baseline Surface Radiation Network of the World Meteorological Organization, World Climate Research Program [25].

Daily (08:00–17:00) and midday (12:00–14:00) solar UV irradiance of a cloudless (clear sky) and of a cloudy day in summer as well as of a cloudless (clear sky) day in winter with high ground reflection (albedo by a snow-covered surface) were selected (Table 1).

**Table 1:** Exposure conditions for each simulation day

Exposure conditions	Season	Calendar day	Daily exposure duration	Midday exposure duration	Weather conditions
1	Summer	17 July 2014	08:00–17:00	12:00–14:00	Cloudless (clear sky day)
2	Summer	10 July 2014	08:00–17:00	12:00–14:00	Cloudy
3	Winter	31 December 2014	08:00–17:00	12:00–14:00	Cloudless with high reflection (albedo) from the ground

#### Sunglasses

**Fig. 2:**
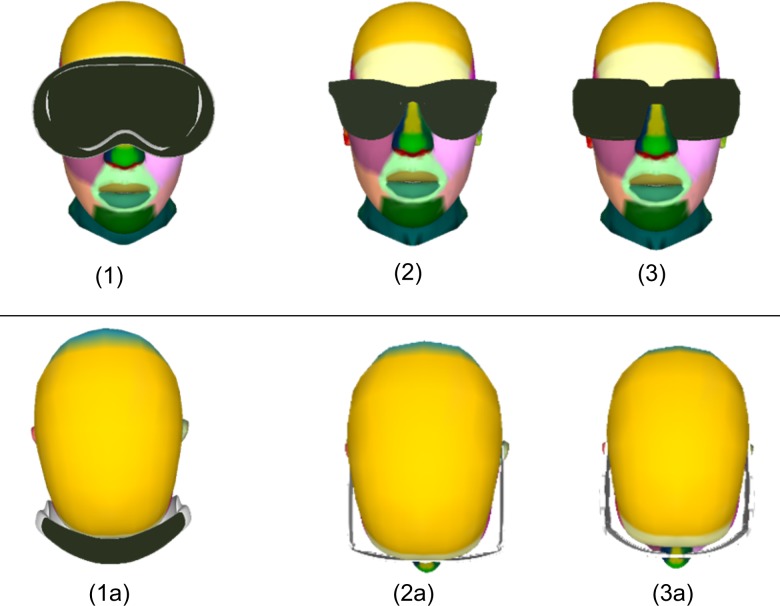
Head with sunglasses (1–3: view from the front, 1a–3a: view from the top, size in cm of sunglasses including frame): (1) goggles (height: 8 cm; width: 15 cm) close-fitting the ocular region, (2) middle-sized sunglasses (height: 5.3 cm; width: 5.7 cm) distant from the eyes, and (3) large-sized sunglasses (height: 5 cm, width: 8 cm) distant from the eyes

Three styles of 3D sunglasses, based on real-life observations, were designed and added to the model (Fig. 2): (1) goggles (size for entire sunglasses: 8 cm high, 15 cm large), close-fitting the periorbital, and ocular skin zones, often used during high sun irradiance situations as being in the snow; (2) middle-sized sunglasses (size for each eye including frame: 5.3 cm high, 5.7 cm large), representing the most commonly used sunglasses in everyday life; (3) large-sized sunglasses (size for each eye including frame: 5 cm high, 8 cm large).

### Output

#### Solar UVR doses and predicted protection factor (PPF)

The estimated cumulative daily and midday solar UVR doses are reported in Joules per square meter [J/m^2^] and given as total or separate UVR component (direct, diffuse, and reflected) doses. The sun protection effectiveness provided by each sunglass style was measured by comparing the total solar UVR dose received (sum of direct, diffuse, and reflected UVR for each skin zone) without and with sunglasses for the same exposure duration. The sun protection effectiveness is expressed as a Predictive Protection Factor (PPF [%]), representing the relative reduction in predicted UVR dose for any skin zone (Eq. 1). The greater the PPF, the higher the relative UVR dose reduction:

1 $${\mathrm{PPF}}[{\mathrm{\% }}] = \frac{{UV_{{\mathrm{withoutprotection}}} - UV_{{\mathrm{withprotection}}}}}{{UV_{{\mathrm{withoutprotection}}}}} \times 100$$

The above equation shows the predictive protection factor (PPF [%]) calculation, estimated for total UVR dose [J/m^2^] received at each skin zone with and without protection.

## Results

### Dosimetry of sunglass lenses

The cumulative erythemally weighted UVR doses measured by dosimetry after five minutes of solar UVR exposure are reported in Table 2. The dose measured without sunglass lenses was 51.1 J/m^2^. By comparison, 5 min outdoor occupational activity in Switzerland (latitude 45.83–47.69, altitude 500–600 m) at midday in summer leads to exposures of 247–495 J/m^2^ [26]. The three sunglass lenses tested showed doses below the threshold limit of <0.5%, i.e., fully absorbed the erythemally weighted UVR independently of their color. These results were considered in the simulations by including the 3D sunglasses (frame and sunglass lenses) as an opaque object.

**Table 2:** Erythemally weighted UVR transmission (%) of three differently tinted non-polarized plastic sunglass lens (category 3) measured with X-2000 personal dosimeter, Gigahertz-Optik (measurement ranges: 165 nW/cm^2^ to 670 mW/cm^2^ with max. 3.3 nW/cm^2^ resolution)

Exposure setup (category 3 sunglasses)	Erythemally weighted UVR transmission measured
Without sunglasses	100% (51.1 J/m^2^)
With a BLUE sunglass lens	< 0.5%^a^
With a GREEN sunglass lens	< 0.5%^a^
With BROWN sunglass lens	< 0.5%^a^

^a^Threshold limit

### Daily exposure

The daily erythemally weighted UVR dose received at facial, periorbital and ocular skin zones without and with sunglasses are given in Tables 3 and 4, respectively, for the three chosen exposure settings (1: cloudless summer, 2: cloudy summer, and 3: cloudless winter with high albedo, see also Table 1) in a “looking-straight ahead” head position. For the unprotected head, highest total doses were estimated at the nose (2195.2 J/m^2^), cheeks (1817.5 J/m^2^), and the cornea (1718.4 J/m^2^) exposed on a cloudless summer day (Table 3). The summer to winter dose ratio on cloudless conditions was highest for the cheeks and nose (UVR dose ratio about 5), and lowest for the periorbital zones (UVR dose ratio about 2.5, Table 3). Total UVR dose received were greater on a cloudless winter day with albedo than on a cloudy summer day (UVR dose ratio range 1.01–1.6), except for the nose which received a slightly lower dose in winter (405.5 J/m^2^ vs 443.7 J/m^2^, respectively). Looking at the contribution of each UVR component (direct, diffuse, and reflected) received during a cloudless summer day, the diffuse UVR dose represent 51–78% of the total UVR dose received. Due to the high albedo, the reflected radiation represented the highest percentage of the total UVR dose received (32–52%) in winter only.

**Table 3:** Daily (08:00–17:00) direct, diffuse, reflected, and total UVR doses [J/m^2^] estimated for unprotected periorbital, ocular, and facial skin zones during: (1) a cloudless summer day, (2) a cloudy summer day, and (3) a cloudless winter day with high reflection from the ground in a “looking-straight ahead” head position

	Exposure		Periorbital skin zones				Ocular skin zones			Facial skin zones	
	Solar UVR component	Exposure condition^a^	Lateral	Lower	Tear duct	Upper	Cornea	White lateral	White medial	Cheeks	Nose
Daily UVR doses [J/m^2^]	*Head without sunglasses*										
	Diffuse	1	698.6	766.1	864.1	641.4	1005.0	773.6	664.9	1052.2	1125.9
		2	280.0	278.0	340.2	275.5	393.5	345.5	270.0	409.0	438.1
		3	129.1	141.6	159.7	118.5	185.7	143.0	122.9	194.4	208.0
	Direct	1	243.7	286.4	623.5	328.3	690.0	288.8	161.7	742.1	1050.7
		2	0.5	0.3	1.0	0.5	1.0	0.4	0.3	1.0	1.6
		3	56.7	68.4	71.6	61.2	90.2	65.5	71.0	64.0	66.6
	Reflected	1	29.6	27.5	21.8	26.8	23.4	28.5	25.2	23.2	18.6
		2	5.9	5.7	4.5	5.6	4.8	5.9	5.2	4.8	4.0
		3	208	193.4	153.1	188.1	164.2	200.5	177.4	163.3	130.9
	Total	1	971.9	1080	1509.4	996.5	1718.4	1090.9	851.8	1817.5	2195.2
		2	286.4	284	345.7	281.6	399.3	351.8	275.5	414.8	443.7
		3	393.8	403.4	384.4	367.8	440.1	409.0	371.3	421.7	405.5

^a^See Table 1 for the specific exposure conditions

**Table 4:** Daily (08:00–17:00) direct, diffuse, reflected, and total UVR doses [J/m^2^] estimated at different skin zones protected by three styles of sunglasses during: (1) a cloudless summer day, (2) a cloudy summer day, (3) a cloudless winter day with high reflection from the ground in a “looking-straight ahead” head position

	Exposure		Periorbital skin zones				Ocular skin zones			Facial skin zones	
	Solar UVR component	Exposure condition^a^	Lateral	Lower	Tear duct	Upper	Cornea	White lateral	White medial	Cheeks	Nose
	*Head with goggles*										
Daily UVR doses [J/m^**2**^]	Diffuse	1	0	0	0	0	0	0	0	362.8	110.5
		2	0	0	0	0	0	0	0	132.0	43.0
		3	0	0	0	0	0	0	0	67.0	20.4
	Direct	1	0	0	0	0	0	0	0	83.6	20.0
		2	0	0	0	0	0.7	0	0	0.7	0.8
		3	0	0	0	0	0	0	0	15.4	15.2
	Reflected	1	0.2	0.7	0.9	0.2	0.3	0.4	0.3	15.7	10.1
		2	5.8	6.2	4.5	5.6	4.9	6.0	5.2	4.8	3.9
		3	1.6	4.8	6.5	1.8	2.2	2.5	4.4	110.5	71.2
	Total	1	0.2	0.7	0.9	0.2	0.3	0.4	0.3	462.1	140.6
		2	5.8	6.2	4.5	5.6	5.6	6.0	5.2	137.5	47.7
		3	1.6	4.8	6.5	1.8	2.2	2.5	4.4	192.9	106.8
	*Head with middle-sized sunglasses*										
	Diffuse	1	149.6	62.7	24.1	59.6	30.5	62.8	88.2	644.0	710.4
		2	58.8	24.0	9.3	2.0	11.5	14.0	44.0	250.0	276.5
		3	27.7	11.6	4.5	11.0	5.6	11.6	16.3	119.0	131.3
	Direct	1	230.3	141.0	37.2	163.8	129.2	222.8	63.0	285.0	651.0
		2	0.5	0.3	0.9	0.5	0.8	0.4	0.3	0.1	1.6
		3	0	0	0	0	0	0	6.8	29.8	46.7
	Reflected	1	11.0	5.8	1.2	2.2	1.0	5.2	2.2	20.0	13.3
		2	2.3	1.2	0.3	0.4	0.2	0.5	0.9	4.0	2.8
		3	77.7	70.5	8.4	14.9	7.3	36.7	15.2	140.2	93.7
	Total	1	390.9	209.5	62.5	225.6	160.7	290.8	153.4	949.0	1374.7
		2	61.6	25.5	10.5	2.9	12.5	14.9	45.2	254.1	280.9
		3	105.4	82.1	12.9	25.9	12.9	48.3	38.3	289.0	271.7
	*Head with large-sized sunglasses*										
	Diffuse	1	41.5	15.6	31.9	53.4	31.7	26.0	33.0	214.9	320.7
		2	0	0.1	0	0	0	0.5	0.4	170.0	374.2
		3	7.7	2.9	5.9	9.9	5.9	4.8	6.0	39.7	59.3
	Direct	1	138.0	96.4	36.3	146.5	102.0	174.8	0	136	578.0
		2	0	0.2	0	0	0	0.1	0.2	0.6	1.5
		3	0	0	0	0	0	0	0	22.9	41.1
	Reflected	1	1.1	0.9	0.5	0.3	0.1	0.3	0.8	10.7	10.4
		2	0	2.6	0	0	0	1.9	2.5	2.3	4.0
		3	7.9	6.5	3.5	1.7	0.4	2.0	5.5	75.3	73.1
	Total	1	180.6	112.9	68.7	200.2	133.8	201.1	33.8	361.6	909.1
		2	0	2.9	0	0	0	2.5	3.1	172.9	379.7
		3	15.6	9.4	9.4	11.6	6.3	6.8	11.5	137.9	173.5

^a^See Table 1 for the specific exposure conditions

Protected by different sunglasses styles, googles blocked almost all UVR received at ocular and periorbital skin zones (Table 4). Protected by middle-sized sunglasses, lateral periorbital (390.9 J/m^2^) and white lateral ocular (290.8 J/m^2^) skin zones received highest total UVR doses during a cloudless summer day. The same skin zones received up to half UVR doses when large-sized sunglasses are worn (lateral periorbital: 180.6 J/m^2^ and white lateral ocular 201.1 J/m^2^).

**Fig. 3:**
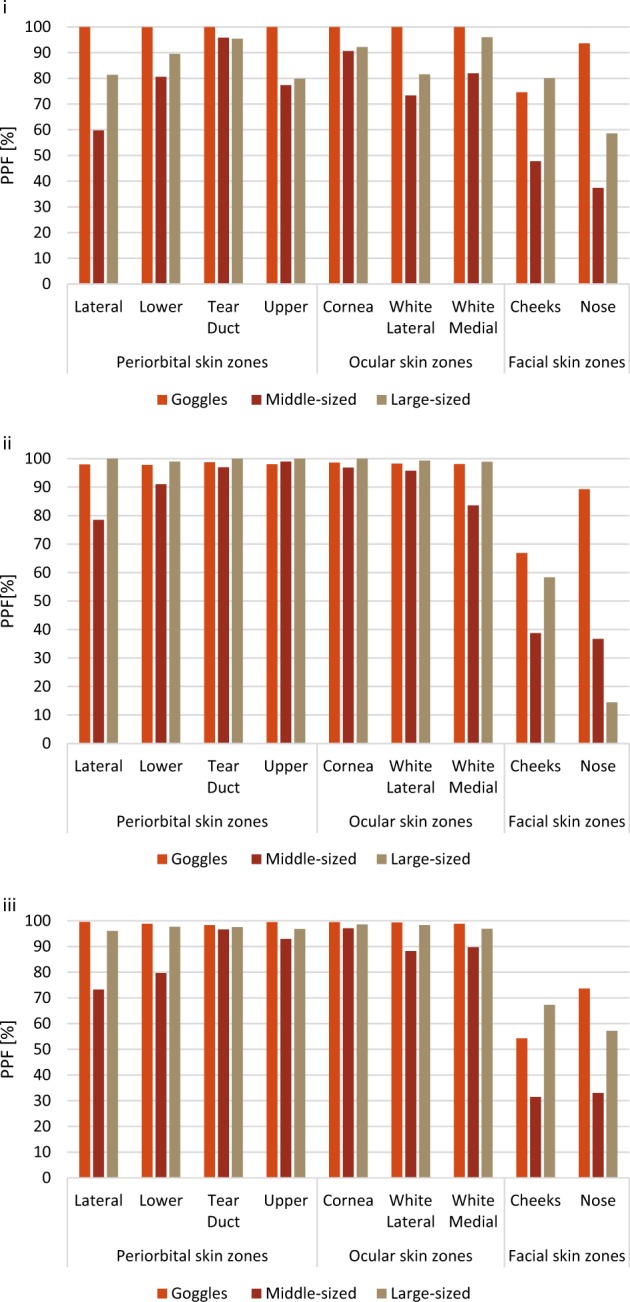
Daily (08:00–17:00) sunglasses’ predictive protection factors (PPF [%]) for periorbital, ocular and facial skin zones for a (i) cloudless summer day, (ii) a cloudy summer day, and (iii) a cloudless winter day with high albedo

The sun protection effectiveness of the tested sunglasses in a “looking-straight” head position is highlighted in Fig. 3 and varied highly between exposure conditions and ocular and periorbital skin zones, except for goggles. Goggles’ PPF values were in all settings for periorbital and ocular skin zones almost constantly 100%, except for the cheeks and nose with lowest UVR protection at the cheeks (PPF 54%) and at the nose (74%) in winter with high reflection form the ground. For all skin zones and exposure conditions, any sunglasses provided at least a PPF of 14%, which was received by the nose protected by a large-sized sunglasses in cloudy summer weather conditions (Fig. 3(ii)). For the periorbital and ocular skin zones, lowest PPF value was estimated at 60% and received by the lateral periorbital skin zone protected by middle-sized sunglasses exposed during a cloudless summer day (Fig. 3(i)). For facial skin zones being protected by middle-sized sunglasses, lowest PPF values were also estimated received at the nose (32%) and the cheeks (31%) exposed during a cloudless winter day with high albedo (Fig. 3(iii)). For the large-sized sunglasses, comparing the various exposure conditions, a PPF of at least 80% was estimated for periorbital and ocular skin zones and of at least 59% for facial skin zones, expect for the nose (PPF 14%) (Fig. 3(ii)). The tear duct and the upper periorbital skin zones were similarly exposed by large- and middle-sized sunglasses during each exposure condition separately. The total doses received with middle-sized sunglasses at lateral and lower periorbital and white-medial ocular skin zones were 2–3 times higher compared to large-sized sunglasses, depending on the exposure condition considered.

**Fig. 4:**
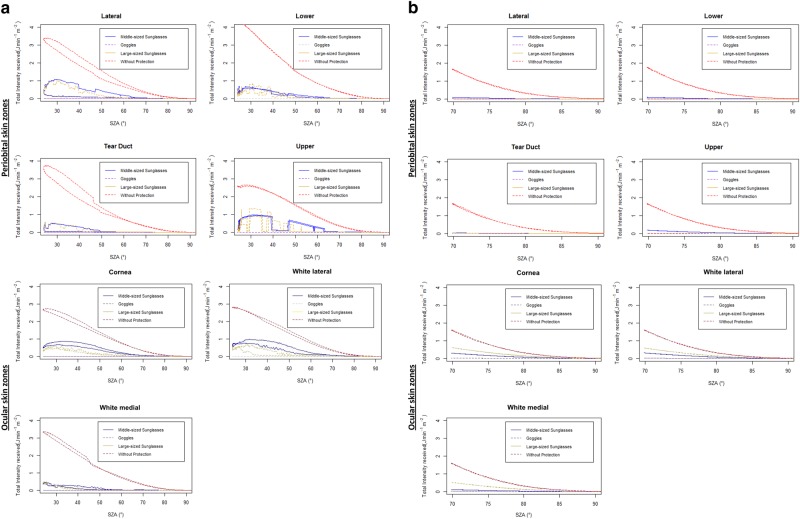
**a** Solar UVR dose variations depending on daily solar zenithal angles (SZA*) for four periorbital and three ocular skin zones unprotected and protected by three different styles of sunglasses during a cloudless summer day (exposure duration: 08:00–17:00). **b** Solar UVR dose variations depending on daily solar zenithal angles (SZA*) for four periorbital and three ocular skin zones unprotected and protected by three different styles of sunglasses during a cloudless day in winter with high reflection from the ground (exposure duration: 08:00–17:00)

The influence of the sun position (solar zenith angle (SZA)) on the total solar UVR received during a summer and winter day are highlighted in Fig. 4b for three ocular and four periorbital skin zones with and without sunglass protection. Each sunglass shows different exposure patterns and peak doses received according to the periorbital and orbital zones considered. During a cloudless summer day, at noon, when the sun is high (SZA low), the dose received is the highest for all periorbital and orbital areas. In winter, especially when the sun is low, the shade provided by sunglasses may not always cover the periorbital and ocular skin zones during the whole day, which means that direct radiation can still reach the eyes. While the solar UVR dose reduction for each zone is important in summer (with a maximal value at noon), the absolute dose reduction in winter is smaller.

### Midday exposure

The midday (12:00–14:00) solar UVR doses received without or without middle- or large-sized sunglass protection were estimated for three head positions (“looking up”, “looking straight ahead”, and “looking down”) and three exposure conditions (Table 1). Highest doses estimates received at the unprotected cornea (908.1 J/m^2^) and nose (811.7 J/m^2^) during a cloudless summer midday in a “looking-up” head position (Table 5). Doses received in a “looking-up” head position were highly reduced by changing head position. For example, at the unprotected cornea the “looking-up versus looking-straight ahead” ratio was 3.3 and the “looking up–looking down” ratio was 8.9 during a cloudless summer midday. With middle-sized sunglasses, highest UVR doses were estimated at the lateral periorbital (105.7 J/m^2^) and the white lateral ocular (133.8 J/m^2^), and periorbital skin zones in the “looking-straight ahead” position on a cloudless summer midday exposure (Table 5). Their UVR doses were reduced by a factor of 1.3–1.6 for the “looking-up” position and by a factor of 2.3–4.7 by a “looking-down” head position. Large-sized sunglasses blocked UVR doses received by periorbital and ocular skin zones in all exposure settings, except lower periorbital, white-lateral and white-medial ocular skin zones receiving in a “looking-straight ahead” head position 93.9, 77.3, and 102.7 J/m^2^, respectively. Compared to middle-sized sunglasses, the nose protected by lage sunglasses received higher doses in a looking-up and looking-straight head position during cloudless summer exposure settings.

**Table 5:** Midday (12:00–14:00) total UVR doses [J/m^2^] estimated at different skin zones protected by middle-sized or large-sized sunglasses taking three different head positions “looking up”, “looking straight ahead” and “looking down” into account

	Simulation		Periorbital skin zones				Ocular skin zones			Facial skin zones	
	Head position	Exposure condition^a^	Lateral	Lower	Tear duct	Upper	Cornea	White lateral	White medial	Cheeks	Nose
	*Head with middle-sized sunglasses*										
Midday UVR doses [J/m^2^]	Looking up	1	76.8	20.2	10.1	19.0	6.6	78.9	8.5	401.1	562.0
		2	20.1	8.2	4.1	6.5	2.7	6.1	14.4	100.8	108.1
		3	35.1	18.4	4.3	8.1	2.8	16.1	25.1	107.1	96.7
	Looking straight ahead	1	105.7	48.2	19.7	69.6	55.5	133.8	10.5	238.1	404.6
		2	20.1	8.2	4.3	6.7	2.7	6.1	14.5	100.9	108.3
		3	35.7	18.1	4.3	9.8	4.2	9.1	19.9	100.1	93.5
	Looking down	1	44.6	18.7	6.4	16.5	8.0	23.6	12.1	154.4	177.4
		2	17.5	7.2	2.5	8.7	3.3	4.8	9.2	51.2	51.8
		3	35.7	17.5	4.3	8.1	3.5	2.8	20.5	107.1	96.7
	H*ead with large-sized sunglasses*										
	Looking up	1	0	0.7	0	0	0	0	1.2	101.8	605.2
		2	0	0.2	0	0	0	0	0.5	22.5	91.6
		3	0	1.1	0	0	0	0.3	0.5	26.3	98.1
	Looking straight ahead	1	0	93.9	0	0	0	77.3	102.7	129.2	505.6
		2	0	36.1	0	0	0	33.2	44.1	50.1	100.8
		3	0	51.1	0	0	0	52.5	39.5	59.1	124.8
	Looking down	1	0	0.5	0	0	0	0.3	0	28.7	122.2
		2	0	0.2	0	0	0	0.1	0	11.5	35.5
		3	0	1.1	0	0	0	0	0.4	22.5	84.3
	*Head without sunglasses*										
	Looking up	1	568.8	650.8	762.6	650.7	908.1	557.1	765.2	766.5	811.7
		2	124.3	135.3	128.2	125.5	156.8	137.7	126.0	155.8	159.1
		3	142.1	145.3	124.2	137.2	150.1	135.1	143.5	148.5	139.1
	Looking straight ahead	1	250.3	242.2	201.6	227.1	271.9	286.5	168.2	495.5	612.8
		2	77.1	80.3	78.3	71.5	97.1	84.4	69.4	115.5	121.1
		3	128.8	130.1	115.3	125.4	104.1	135.1	116.2	142.3	132.6
	Looking down	1	102.1	91.5	78.5	75.1	101.1	102.7	60.4	222.8	269.4
		2	40.3	36.2	30.2	28.1	40.5	40.8	23.3	77.1	79.5
		3	112.2	111.3	98.5	107.5	121.4	117.1	97.3	130.7	124.3

^a^See Table 1 for the specific exposure conditions

Midday sun protection effectiveness of sunglasses (PPF values) is affected by the head position while wearing middle-sized sunglasses (Annex: Suppl Figure 5a), and to a lesser extent when wearing large-sized sunglasses (Suppl Figure 5b). PPF values of different head positions only vary slightly between the different exposure conditions for large-sized sunglasses (Annex: Suppl Figure 5b), except for lower periorbital and white lateral and medial ocular skin zones, as well as the cheeks and the nose.

## Discussion

The prediction of the facial, periorbital and ocular sun protection effectiveness provided by different styles of sunglasses enables to quantify direct, diffuse and reflected UVR doses received by the eye and its surrounding skin zones. Experimental results have shown that the tested plastic sunglass lenses are fully absorbing UVR and can thus be considered as opaque surfaces. High UVR doses were received at unprotected skin zones for all considered midday and daily exposure conditions. Sunglasses reduced UVR doses received. Besides close-fitting sunglasses as googles, no sunglasses fits all situations. The dose estimates accounted for influencing factors as sunglasses geometry and wearing position, the distance between nose bridge and forehead, the skin zone, the sun and head position, the orientation toward the sun, the cloud cover and the reflection from the ground. Our results also highlight a high dependency between the sun protection effectiveness and the geometry of sunglasses.

The most sun protective sunglasses in all exposure conditions were close-fitting goggles, blocking UVR from all directions. The estimated UVR doses received by skin zones protected by middle or large-sized sunglasses highly differed and strongly depended on environmental conditions. Ocular doses received with commonly worn middle-sized sunglasses were greater compared to other tested sunglasses. This confirms that indirect light pathways do contribute to the eyes’ sun exposure, indicated by high diffuse and reflected UVR dose [19]. Indeed, middle-sized sunglasses highly protect from direct UVR but sparely from ground-reflected UVR. The contribution of reflected UVR was predicted by Sliney et al., but the impact of sunglasses shape on protection from reflected UV has not been modeled [4]. Importantly, in winter, the reflected UVR dose remains high even when sunglasses are worn.

Daily cumulative doses received by periorbital and ocular skin zones protected middle- or large-sized sunglasses exceeded the 1.2 SED (120 J/m^2^ erythemally weighted) recommended threshold in cloudless summer exposure conditions, except for the tear duct (Table 4, for both sunglasses) and the white-medial skin zone (only for large-sized sunglasses) [27]. Additionally, the head position toward the sun bear an important influence on UVR dose received, especially for middle-sized sunglasses (Table 5). While a “looking-down” head position efficiently blocked UVR, the frequent “looking-straight ahead” head position was the least sun protective for ocular and periorbital skin zones as the sun rays could circumvent the sunglasses. Thus the development of sunglasses providing a tighter fit to the head’s geometry may provide higher PPF values.

Our study shows that different representations of the UVR doses received are fundamental for effective sun protection from different perspectives. From a medical point of view, correlations between eye diseases and sun exposure are difficult to establish because the dose of light received is not estimated accurately. The absolute dose estimates may help identify underestimated exposures conditions. Interestingly, our result show that the cornea, the cheeks and nose receive the highest UV doses during a cloudless summer day. From a public health perspective, knowledge of the doses received according to UVR component (direct, diffuse, and reflected) and exposure settings, is pivotal to identify various sunglass shapes’ sun protection deficiencies. From a more global perspective, the relative UVR dose reduction (PPF) provided by sunglasses is not necessarily related to the risk of UVR-induced ocular diseases. It guides the choice toward efficient UVR blocking glass, but does not provide information regarding the best sunglass shape and style depending on the exposure situation and the face shape.

This study presents an elegant and quick method to quantify erythemally weighted UVR doses received at various periorbital and ocular skin zones that are difficult to assess and challenging for dosimetry. The 3D simulation generates detailed exposure data about the ocular regions, entails a fine computation of the incoming radiation, and conveys information on UVR intensities and incidence angle. Our finding are in line with the few published dosimetry studies. Even using specific action spectra for different eye diseases (i.e., cataract, photokeratitis, photoconjunctivitis), these studies found maximum doses for lower SZA (higher solar elevation angles) in relation with different mannequin orientation or regression models underlying the effect of the diffuse radiation on the eye since it is incident from all directions, and showing some bimodal distributions with peak values in the morning and in afternoon such as in the case of the upper periorbital skin zone (Fig. 4a) [28–33].

Even if goggles are considered as most sun protective and these sunglasses might not be commonly used except for specific sport or occupational activities, our study results underline the importance of adapting sun protection and prevention messages to exposure conditions. Referring to the results reported for the unprotected skin zones (Table 3), the importance of the geometrical factors of the anatomy of the periorbital skin zones needs to be underlined, as the upper lid and brow lids’ position highly reduce the exposure of the cornea to direct UV rays in a “looking-straight” head position [34]. This is concomitant with Hu et al. findings, who observed sharp increase or decrease in irradiance measurements when varying rotation and elevation angle of an exposed manikin [28]. Additionally, the distance between nose bridge and sunglasses (Fig. 2, view from the top) needs to be considered, which may explain surprising finding of higher doses estimated for the nose protected by large-sized sunglasses compared to middle-size sunglasses (Table 5). Our study’s large-sized sunglasses are less close-fitting to the nose bridge and to a lesser extent block direct and reflected radiations reaching the nose, especially in a “looking-up” or “looking-straight” head position. Adjusting sunglasses’ shape to individuals’ anatomy and environmental and outdoor activities conditions should be the next challenge in improving eyes UVR protection.

This study’s results have some limitations. The head morphology implemented in SimUVEx includes neither eyebrows, eyelids, cilia nor scalp hair. Further, our predictions assume no UV protection from other sources such as a hat, natural or artificial shade, side shields, or an umbrella. Both these limitations lead to an overestimation of the absolute UV dose reaching the eye. However, this limitation does not affect our comparison of doses received with and without sunglass-wear, as the UVR reduction provided by anatomical or additional protection means should be the same in both cases. Regarding the input data, the erythemally weighted spectrum used in this exposure study does not separate UVA from UVB of the solar radiation to better understand the absorption and transmission of each ocular skin layer. While the erythemal action spectrum is widely used in photobiology, an action spectrum more specific to the ocular damage, such as the photokeratitis or cataract spectrum would have been more appropriate. However, it should be noted that these spectrums are very close. Measurements of the daily erythemally weighted and photokeratitis doses in different regions and period of the year were performed by Grifoni et al. [35]. It was shown that, for a same region, the dose ratio between the two action spectrum were consistent over the year and typically in the range of 1.2–2.0. Lastly, SimUVEx handles direct illumination aspects only, meaning that indirect illumination provided by the reflection of the sunglasses frame is not taken into account. Therefore, our estimated doses pertain to sunglasses with a non-reflecting (mat) frame material. As this study includes preliminary 3D sunglass developments, their fit on the 3D head morphology needs further graphic improvements to unrealistic UVR doses received as for reflected UVR results for the eyes covered by goggles or large-sized sunglasses with no close-fitting nose bridge.

This model could be used to support the development of new sunglass styles by predicting the protection effectiveness early and in silico. In addition, the external eye-exposure information generated by SimUVEx could be coupled with an intraocular eye model in order to predict retina exposure in various environmental conditions. This work warrants further developments and studies to better understand the effectiveness of sunglasses side shields, the combination of various sun protection means as hats, scarfs, neck flags and shade structures. 3D modeling permits to estimate solar UVR doses received at highly sensitive small skin zones such as the eyes, chronically exposed and rarely assessed, and to better understand dose–response relationships of solar UVR-induced ocular diseases.

## Electronic supplementary material

Supplementary Figure 5
