# Supplementary material for: Sun exposure to the eyes: predicted UV protection effectiveness of various sunglasses
Source: J Expo Sci Environ Epidemiol. 2018 Oct 31;29(6):753–64. doi: 10.1038/s41370-018-0087-0 (PMC6803516; doi:10.1038/s41370-018-0087-0)
Supplement: Supplementary file 1 — Supplementary Figure 5 [file 41370_2018_87_MOESM1_ESM.docx]

i

ii

iii

**Figure 5a:** Midday (12:00-14:00) sunglasses’ Predictive Protection Factor (PPF [%]) for periorbital, ocular and facial skin zones exposed during a (i) cloudless summer day, (ii) a cloudy summer day or (iii) a cloudless winter day with high albedo protected by **middle-sized sunglasses** in three different head positions.

i

ii

iii

**Figure 5b:** Midday (12:00-14:00) sunglasses’ Predictive Protection Factor (PPF [%]) for periorbital, ocular and facial skin zones exposed during a (i) cloudless summer day, (ii) a cloudy summer day or (iii) a cloudless winter day with high albedo protected by **large-sized sunglasses** in three different head positions.
